# A workshop on leadership for senior MD–PhD students

**DOI:** 10.3402/meo.v21.31534

**Published:** 2016-08-05

**Authors:** Catherine B. Meador, Bobak Parang, Melissa A. Musser, Rachana Haliyur, David A. Owens, Terence S. Dermody

**Affiliations:** 1 Medical Scientist Training Program, Vanderbilt University School of Medicine, Nashville, TN, USA; 2 Owen Graduate School of Management, Vanderbilt University, Nashville, TN, USA; 3 Department of Pediatrics, Vanderbilt University School of Medicine, Nashville, TN, USA; 4 Department of Pathology, Microbiology, and Immunology, Vanderbilt University School of Medicine, Nashville, TN, USA; 5 Elizabeth B. Lamb Center for Pediatric Research, Vanderbilt University School of Medicine, Nashville, TN, USA

**Keywords:** MD–PhD curriculum, leadership, conflict management, student-led program design, case-based instruction, MSTP

## Abstract

Leadership skills are essential for a successful career as a physician-scientist, yet many MD–PhD training programs do not offer formal training in leadership. The Vanderbilt Medical Scientist Training Program (MSTP) previously established a 2-day leadership workshop that has been held biennially since 2006 for students in the first and second years of the graduate school portion of combined MD and PhD training (G1/G2 students). Workshop attendees have consistently rated this workshop as a highly effective experience. However, opportunities for structured training in leadership competencies during the subsequent 3–5 years of MD–PhD training are limited. Given the success of the G1/G2 leadership workshop and the need for continuity in this model of leadership training, we developed a half-day workshop for MSTP students in the clinical years of medical school (M3/M4 students) to foster continued training in leadership. Our workshop curriculum, based in part on original cases drafted by Vanderbilt MSTP students, provides concrete strategies to manage conflict and navigate leadership transitions in the physician-scientist career path. The curriculum emphasizes both short-term competencies, such as effective participation as a member of a clinical team, and long-term competencies, such as leadership of a research team, division, or department. Our inaugural senior leadership workshop, held in August, 2015, was judged by student participants to be well organized and highly relevant to leadership concepts and skills. It will be offered biennially in our training curriculum for M3 and M4 MSTP students.

Institutional cultivation of strong leadership competencies has been acknowledged as a necessary element in the training of physician-scientists (1, 2). Specifically, the National Academy of Sciences has recommended the broadening of educational opportunities for postdoctoral trainees in laboratory management and mentoring to support timely and successful establishment of independent careers (1). Additionally, members of healthcare teams with strong leaders are more likely to report satisfaction and less likely to develop burnout (3). However, while the need for leadership development in physician-scientist training is widely acknowledged, the optimal mechanism and timing of leadership training remains an open question.

Several healthcare organizations and academic centers have established leadership programs in response to the need for more formal leadership development of faculty and trainees (1, 4–6). Such programs and published materials for postdoctoral fellows and junior faculty members increasingly provide opportunities to learn or refine leadership skills such as those in communication, collaboration, feedback, mentoring, and management. These efforts positively contribute to effective leadership of clinical and research teams, achievement of tenure, and scientific publications (1, 6–8). However, such programs are not yet standard practice in the predoctoral training of physician-scientists.

Completion of medical school and graduate school requirements provide important experiential learning of leadership skills. We previously hypothesized that MD–PhD training programs (Medical Scientist Training Programs, or MSTPs) provide an ideal environment for incorporation of formal leadership development, particularly given that mission statements of multiple programs nationwide explicitly describe a commitment to training leaders in investigative medicine. For the past decade, we have sought to design and implement leadership programming for MD–PhD students in the Vanderbilt MSTP to fill these critical gaps in medical and research training.

## Vanderbilt MSTP Junior Leadership Workshop

**Fig. 1:**
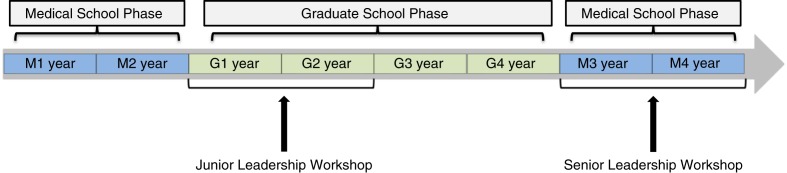
Schematic of MSTP leadership workshop timing within the MD–PhD training curriculum. Since 2006, the Vanderbilt Medical Scientist Training Program (MSTP) has hosted a leadership workshop biennially for MD–PhD students in their first and second years of graduate school (G1/G2; ‘Junior Leadership Workshop’). The MSTP Senior Leadership Workshop, held for the first time in August 2015, was attended by senior, clinical-phase Vanderbilt MD–PhD students enrolled in the final two years of medical school (M3/M4). M, medical school year; G, graduate school year.

In 2006, the Vanderbilt MSTP instituted a biennial 2-day leadership workshop for MD–PhD students in their first and second years of graduate school (G1/G2; Fig. 1) (9). The goal of implementing this junior leadership workshop was to provide formal training in leadership during MD–PhD training. In the first 2 years of graduate school, students learn firsthand about team dynamics and mentoring styles as they gain experience as members of investigative teams. The junior leadership workshop was designed to provide structured programming to complement student experiences at this stage of training, allowing time to apply new leadership competencies during graduate school. This workshop has been an integral part of our MSTP curriculum for 10 years and employs an original design continually modified by a joint student–faculty committee responsible for planning and execution of the workshop. The junior leadership workshop employs a case-based learning format to cover a variety of topics including leadership styles, recruitment and retention, motivation, coaching, and feedback and has been consistently rated by MSTP students as a highly effective learning experience.

## Vanderbilt MSTP Senior Leadership Workshop

While a one-time workshop is useful for exposure to concrete, learnable leadership skills, true leadership development requires time. More importantly, it requires application of new skills, revisiting of previously learned leadership concepts, and experience in multiple different professional contexts as one matures (10, 11). Fortunately, the 7–8 years that each Vanderbilt MSTP student will spend in MD–PhD training provides substantial time for the student to apply and reflect on learned leadership competencies from the junior leadership workshop. However, students spend those years rotating between clinical and research-based academic endeavors, and the interpersonal dynamics and hierarchical nature of teams in research and clinical settings can vary significantly. As such, the leadership competencies required for navigating these situations are unique.

Given the success of our junior leadership workshop and the opportunity for subsequent leadership development during MD–PhD training, we hypothesized that our MD–PhD trainees would benefit from reinforcement of important leadership competencies in the form of a follow-up workshop during their final clinical years of medical school (M3/M4; Fig. 1). Therefore, we developed a workshop focused on topics relevant to senior MD–PhD students preparing to enter internship and residency and begin a career in investigative medicine.

We formed a committee that included MSTP students, the MSTP director, and a faculty member from the Owen Graduate School of Management with expertise in organizational behavior to plan and facilitate the inaugural senior leadership workshop. While we incorporated programmatic expertise gained from hosting previous junior leadership workshops in planning this new curriculum, our planning committee was a distinct entity, independent of the planning of the junior leadership workshop. Our intent for the senior workshop was to design a half-day curriculum that accomplished two goals: 1) provide content that served as a refresher and follow-up of experiences of the junior leadership workshop and 2) use real-life case scenarios relevant to senior MD–PhD (M3/M4) students. In this way, we challenged senior students to think beyond previously attained leadership competencies and consider additional leadership challenges inherent to the physician-scientist career, both from short- and long-term perspectives.

## Content survey

In contrast to our junior leadership workshop, which has consistently been designed as a 1–2 day event, the limited time available for senior students required a more focused selection of content for the senior leadership workshop. To identify topics of greatest interest to senior MD–PhD students, we administered a survey to rising M3 and M4 Vanderbilt MSTP students (anticipated workshop attendees) as well as graduating M4 students. In this survey, we provided a list of leadership competencies, some of which were covered in the junior leadership workshop, and asked students to select three to four topics from this list thought to be most important. The list of competencies included conflict management, feedback, coaching, expectations, motivation, directing group meetings, communication styles, effecting change, and building a team. We also listed a few potential scenarios for case studies and asked students to select three that were thought to be most relevant. Finally, we provided additional response space in which we encouraged students to suggest potential case studies from their experiences in investigative or clinical teams.

The two leadership competencies most frequently selected by students as topics warranting further discussion were conflict management and building a team. In addition, among the potential case scenarios most frequently selected by students were 1) management of administrative, clinical, and research time by an assistant professor and 2) establishment of priorities by a department chair. Based on this feedback, we chose two main areas of focus for our senior leadership workshop. First, we designed a case study focused on conflict management within a clinical team. Conflict management was a topic of study for the junior leadership workshop, and given the responses from students about their continued interest in this topic, we concluded that it would be valuable to revisit. Furthermore, since previous case studies in the junior leadership workshop had focused primarily on conflict management within a research team (as junior leadership workshop attendees are in the early years of graduate school), we thought it would be useful to address conflict management in the clinical setting. Second, we designed a case study focused on the challenges of transitioning between leadership positions within academic medicine. This case study would jointly address building a team and managing administrative, clinical, and research priorities as a faculty member and leader.

## Workshop curriculum

### Pre-workshop survey

One month prior to the workshop, we distributed a survey to workshop attendees to obtain participant-level information relevant to the planned curriculum. The first section of the survey was a conflict management style self-assessment (12), which assigned relative rankings of each individual's conflict management style in the following categories: collaborating, competing, avoiding, harmonizing, and compromising. The goal of this self-assessment was to prime workshop participants for discussions about conflict management by facilitating self-reflection before the cases were considered by the group. We thought that self-reflection about conflict management would be especially important given that the workshop was confined to a half day, eliminating the opportunity to introduce initial concepts, allow for a period of reflection, and re-open the discussion the following day. In addition, the de-identified results of these conflict management style assessments were correlated with gender and medical specialty of interest, providing cohort information useful to the workshop facilitator and of interest to participants when presented during the didactic portion of the workshop.

The second section of the pre-workshop survey was designed to elicit perspectives about leadership from student attendees. Specifically, we asked students to list the top three most and least effective leadership qualities they had observed thus far in their academic careers. We then asked students to list desired leadership positions in their future careers. By asking students to record these observations and career goals in advance of the workshop, we hoped to facilitate discussion among peers and with faculty facilitators about plans to achieve stated goals, potential barriers to success, and rationale for deciding which professional endeavors to pursue when given the opportunity. We thought that this stage of training, when students were thinking most intently about post-graduation plans and further training in medicine and science, would be an ideal time for encouraging students to thoughtfully consider future leadership opportunities and how to achieve them.

### Case studies

To facilitate discussion during the workshop about the selected curriculum topics, we drafted two original case studies. Content areas for the cases were identified by workshop attendees to be interesting and relevant to their career aspirations. Discussion of these cases comprised the majority of our workshop curriculum. For each case, workshop participants were assigned to groups of four to five for initial discussion. The students then re-convened as a group (total 23 students) to compare conclusions and points of interest with their peers, the workshop facilitator, and additional content experts.

The follow-up sessions after each small-group case discussion were formatted as discussion-based lecture material to expand on the students’ preexisting knowledge base, incorporate results from the pre-workshop self-assessment exercise, and apply theoretical concepts back to the case scenarios. By incorporating these didactic sessions after the initial case discussions, we used the ‘challenge cycle’ of learning, in which students had an opportunity to probe their initial understanding of the topics at hand before engaging in a didactic learning format. The challenge cycle is a well-established teaching modality that we have successfully employed in previous leadership workshops (9, 13). Overall, the goal of these case-based discussions was to encourage context-based learning relevant to MD–PhD students’ future career endeavors.

### Case #1: ‘Bottom of the Heap’

Our first case, entitled ‘Bottom of the Heap’, focused on conflict management within a clinical team (Appendix, Box 1). We presented a scenario in which a new intern working in an emergency department disagrees with other members of the medical team about the appropriate plan of care for one of her patients. She is forced to navigate a hierarchical team of co-residents, attending physicians, and consulting physicians to resolve the disagreement and provide optimal care. She also struggles with how to respond to the concerns and questions of the patient's family, given that the situation is largely outside of her direct control. In the discussion questions following the case, we encouraged students to consider the conflict management styles of each case character and provide potential avenues that could be used by this intern to reach a resolution for the described scenario. Following the case discussion, our workshop facilitator led a didactic session focused on conflict management, incorporating responses collected in the pre-workshop survey and encouraging students to reflect on their own conflict management styles. In sum, the purpose of this first case discussion was to present a realistic scenario that reflects the type of situation students will navigate as new resident members of clinical teams. The goal of the discussion was to provide students with tools to approach conflict in a more confident and prepared manner early in their professional careers.

### Case #2: ‘On the Road Again’

Our second case, entitled ‘On the Road Again’, focused on transitions between leadership positions in academic medicine (Appendix, Box 2). We described a mid-career physician-scientist who is recruited to a new institution to take a position directing a large clinical division. The case describes the challenges faced by this individual as she decides whether to take the position, considers strategies for retaining and recruiting members of her research team, and learns to balance administrative, clinical, and scientific aspects of her new position. Discussion questions for this case also focused on these issues, prompting students to consider strategies for making transitions between leadership positions in academic medicine. We thought that this case was especially relevant given that, according to our survey results, 83% (19/23) of our workshop participants have aspirations to become a division director or department chair at some point in their careers.

For Case #2, our follow-up discussion extended into an informal reception and dinner session of the workshop and included prominent Vanderbilt faculty members (including two current division directors and a department chair) in a panel discussion. The panelists were asked to discuss challenges faced and decisions made while serving in various leadership positions over the course of their careers. The panelists then answered questions submitted by the workshop participants. As such, the panel discussion provided an opportunity to learn from institutional leaders about a variety of topics of interest from the afternoon workshop, including real-world conflict management and leadership strategies.

## Evaluation

### Post-workshop evaluation

Immediately following the workshop, we elicited feedback from student participants in the form of a written evaluation (Table 1). Similar to the evaluations for our MSTP Junior Leadership Workshop (4), this evaluation included one section of Likert-scale questions, in which students were asked to indicate their opinion about the organization, execution, and relevance of each section of the workshop (1, strongly disagree; 5, strongly agree). The second portion of the evaluation consisted of free-response questions soliciting information from students about the utility of the workshop content, the most valuable aspects of the workshop curriculum, and areas for improvement. These questions included: Was the workshop well balanced in terms of presenting didactic information versus encouraging discussion? In what way, if any, did this workshop affect your personal plans for leadership development? Is there anything you heard today that will affect how you approach conflict in the future? What is the most valuable skill or concept that you gained from the workshop? Were there any issues or skills you wish the workshop had covered that it did not?

**Table 1:** Likert-scale evaluation responses of 2015 MSTP Senior Leadership Workshop by student participants^a^

Workshop activity	Well organized and executed?	Relevant to leadership concepts and skills?
Conflict Management Styles Quiz (pre-workshop survey)	4.47	4.56
Case Study #1: ‘Bottom of the Heap’	4.64	4.59
Didactic Session on Conflict Management	4.53	4.29
Case Study #2: ‘On the Road Again’	4.41	4.53
Dinner Discussion: Leadership in Academic Medicine	4.73	4.73

a Data represent mean responses using a five-item scale (1, strongly disagree; 5, strongly agree; *n*=17)

On our Likert-scale evaluation questions, the majority of students selected either ‘strongly agree’ or ‘agree’ when asked whether each element of the workshop was well organized, well executed, and relevant to their understanding of leadership concepts and skills (Table 1). The two most highly rated workshop activities were the case study on conflict management (Case #1: ‘Bottom of the Heap’) and the after-dinner panel discussion with Vanderbilt faculty leaders about academic leadership.

When asked whether the balance between didactic learning and opportunities for discussion was appropriate, all student participants answered in the affirmative, many repeatedly noting throughout the evaluation that the discussion portions were a strength and highlight of the workshop experience. When asked about the effect of the workshop on students’ personal plans for leadership development, students replied with such statements as: ‘It made me think of what I want my career to look like in 20 years – during a time when it's hard to think beyond residency’, ‘It provided clarity on what the tasks and expectations are for leadership positions in academic medicine. It also put leadership styles and conflict management skills into a more structured, comprehensive context’, and ‘[It] affirmed my desire for an academic leadership position’. Two of 13 respondents noted that it did not significantly change their thinking but merely affirmed their previous career plans.

When asked whether there was anything the students heard during the workshop that will affect how they approach conflict in the future, students replied with such responses as: ‘… Primarily, to gauge where I'm coming from, where the other person is coming from, and then act. And don't be afraid to ask for help and advice’, and ‘It highlighted the value of stepping back from tense conflict situations and resolving issues in a constructive, mutually beneficial way’.

One of the most frequently cited areas for improvement in future workshops was the suggestion that we minimize the small group (four to five students) discussion of each case study and maximize the large group discussion (~20 students) with our workshop facilitator. In addition, two students noted that the timing of this workshop could have been improved; specifically, an M4 student suggested that August during the fourth year of medical school was an especially busy time. While later in the fall semester would likely lead to a lower turnout of M4 students due to away rotations and residency interviews, it might be preferable to move the workshop to the spring semester in future iterations. Notably, we were able to achieve 75% (22/29) attendance of the M3 and M4 MSTP classes based on extensive consultation of medical school calendars and restriction of the workshop to a half-day Saturday experience.

### Follow-up survey (6 months later)

To determine whether the workshop curriculum influenced student attitudes and behaviors about conflict resolution and leadership development beyond the workshop interval, we surveyed workshop attendees 6 months after the workshop was completed (Table 2). We asked students to reflect on personal and professional experiences during the 6 months following the workshop and assess any changes in attitudes or behaviors about the leadership competencies addressed.

**Table 2:** Six-month follow-up survey administered to the participants of 2015 MSTP Senior Leadership Workshop

Survey question		Frequent themes in response
Please describe in what way, if any, the following have changed as a result of your participation in the MSTP Senior Leadership Workshop:	Your *attitude* about conflict in personal or professional situations	Less likely to avoid conflict More thoughtful about others’ perspectives and methods of managing conflict
	Your *behavior* in conflict situations	More proactive in engaging in conflict resolution Seeking the underlying cause of conflict, rather than taking it personally
	Your *attitude* about work–life balance	Balance is important in order to be successful Balance only gets more difficult as a career progresses, start forming habits now
	Your *decisions* about work–life balance	More thoughtful about how to spend time professionally and personally
What additional behavioral or attitudinal changes, if any, have you made since the workshop in response to what you learned?		More proactive about seeking guidance
		More aware of each role on the team More thoughtful about gender equality in science and medicine
What are the three most critical skills, behaviors, or insights from the workshop that have affected your professional or personal life?		Skills in conflict resolution Strategies for recruitment of laboratory personnel Effective communication

In response to this survey, students consistently noted that, as a result of workshop discussions, they were more accepting of conflict as a normal occurrence in the workplace and more likely to engage, rather than to avoid, conflict in an attempt to resolve conflict as it becomes apparent (Table 2). Additional behavioral changes that students recognized and attributed to the workshop experience included being more proactive about seeking guidance, more attentive to gender equity in academic medicine, and more likely to engage in casual conversations with peers about development of leadership skills.

## Discussion

The inaugural MSTP Senior Leadership Workshop was an effective addition to the Vanderbilt MSTP curriculum. Evaluations of the initial offering of the workshop indicate that the workshop was well received by students and that the workshop curriculum influenced student thinking about leadership development. Similar to our experience with the MSTP Junior Leadership Workshop (9), we found that the curriculum served not as a strict template but as a starting point for a variety of engaging, and at times impassioned, discussions about topics of interest to the group. For example, in addition to the discussion questions provided for each case, additional topics raised by the students included the importance of different types of leadership styles, appropriate communication strategies for a leader to inform team members when planning to change institutions, the role of gender in conflict and hierarchy on clinical teams, and a physician-scientist's relative obligation to his or her various scientific, clinical, and administrative responsibilities.

We can make three general conclusions from the feedback obtained from the first cohort of workshop attendees. First, our experience with this group demonstrated that discussion of conflict management was a useful aspect of the curriculum. The feedback we received from students indicated that this was an appropriate time in their training paths for a refresher discussion of this topic, which was introduced during the MSTP Junior Leadership Workshop. Second, inclusion of Vanderbilt leaders in academic medicine in the workshop curriculum was beneficial for students as they explored their own leadership development, career aspirations, and balance of personal and professional responsibilities. Third, we will reevaluate the most appropriate timing for this workshop during the academic year, given that scheduling for students in the clinical years of medical school can pose difficulties.

We have continued our successful model of involving students in curriculum design and execution of this workshop, and we intentionally included junior students who will assume leadership roles and recruit new student members to the planning committee for the next version of the workshop in academic year 2017–2018. Over the next several years, we will continue to revise the workshop curriculum based on student feedback. From a long-term perspective, we will follow the careers of the workshop participants to understand more fully the effect of our leadership workshops on the professional aspirations and achievements of MD–PhD students.

## Appendix

Box 1 Case Study #1: Bottom of the Heap On conflict management and dynamics within a clinical team Joy Brady is a new emergency medicine intern who graduated from medical school at the top of her class. She is 4 months into her internship and starting to feel more confident handling the workload and making independent decisions about managing patients. A little after 11 pm on her third night shift in a row, Joy was asked to evaluate a 12-year-old boy with abdominal pain. After a brief look at the vital signs (temperature 100.4°F, pulse 110/min, BP 92/64 mm Hg, RR 16/min, O_2_ saturation 99% while breathing room air), Joy headed down the hall to meet the patient. The child was in the room with his mother, who had brought him to the ED for evaluation of abdominal pain. Tearful and quiet, he told Joy that his stomach had been hurting all day. His mother told Joy that her son went to the school nurse at lunch time complaining of abdominal pain and decreased appetite. After coming home from school, he mostly stayed in bed but was in too much pain to sleep. His mother tried to feed him soup around 3 pm, but he vomited shortly afterwards and hadn't eaten since. When he complained that the pain was getting worse, she brought him to the ED. His mother reported that the child had infrequent episodes of abdominal pain in the past associated with constipation, but nothing like this. He wasn't taking any other medications and hadn't been in contact with friends or family members who were sick. His mother thought his forehead felt hot, but she didn't have a thermometer to check his temperature. When asked whether the pain had changed locations during the day, the boy just shrugged his shoulders. On physical exam, the child was tender to palpation in the right lower quadrant and had some rebound tenderness and voluntary guarding. Joy also noted a positive Rovsing's sign. Joy told the child and his mother that she would be back shortly, and she left the room to place some orders. Thinking that appendicitis should be at the top of her differential diagnosis list, she ordered a 20 ml/kg fluid bolus, 0.1 mg/kg morphine for pain, 4 mg ondansetron (Zofran) for nausea, a CBC panel, and an ultrasound. The CBC showed a white blood cell count of 12,800 cells/microliter (74% neutrophils, no bands), hemoglobin 14 g/deciliter, and platelets 280,000/microliter. The ultrasound showed a non-compressible blind-ending appendix measuring eight millimeters in diameter, suggestive of appendicitis. However, there was no evidence of surrounding inflammatory changes. Joy knew that a CT scan would provide more anatomic detail, but she also knew the guidelines about limiting radiation exposure in children. Besides, the physical exam findings were textbook for appendicitis. Joy found Marissa Maynard, her upper-level resident, and told her about her plan for the patient, saying that she would consult surgery to evaluate the child for an appendectomy. Marissa agreed with the plan and also agreed that a CT scan would be inappropriate in this case. Joy put in the consult to surgery and shortly afterwards received a call back from George Hall, the senior surgery resident on call. George asked impatiently whether this was an emergent situation, saying that he was headed to the OR for an aortic aneurysm repair. Joy quickly explained the case, her diagnosis, and her plan for the patient. George replied that he did not have time to examine the patient until after the aneurysm repair and asked if she was sure about her physical exam findings. ‘Less than 10% of patients with this constellation of findings actually have appendicitis, anyway’, George said impatiently. Joy knew that this simply was not true, and she had been sure about this case and the plan for the patient. Simultaneously irritated at George and fighting the urge to lose confidence in her own diagnostic skills, Joy tried not to raise her voice as she asked how soon George or someone from his team could come see the patient. George replied, ‘Look, I'll come see the kid as soon as I can. It won't be until after we're finished in the OR. This could be anything, and I'm not worried about this patient tonight. If you're so sure this patient needs to be admitted, send him to the pediatrics service for serial abdominal exams. Who's the attending down in the ED right now, anyway? Is anyone else involved in this case?’ Joy was frustrated and angry. She found Marissa to ask what she should do. Joy clearly couldn't admit the patient for surgery without George's approval. Since she had already given the child pain medication, whenever the surgery team did make it down to come see him, they may not be able to reproduce her original exam findings. Should she change course and just admit the boy to the pediatrics service and let them deal with it? Marissa told her that there was no reason to raise any further issues and that she should just stop worrying about it and move onto the next patient. One of the ED nurses approached Joy to tell her that the boy's mom was concerned about how long they might have to wait before the surgery team could see her son. ‘What should I tell her?’ she asked Joy.
Discussion questions:

1. What are the conflicts in this scenario?
2. What is the source of Joy's frustration? What is the source of George's frustration?
3. How did each of the main characters approach this conflict?
4. Who is available to provide Joy with assistance?
5. How should Joy proceed?
6. How did the genders of Joy and George contribute to this conflict?

Box 2 Case Study #2: On the Road Again On leadership transitions in the physician-scientist career track Mary O'Rourke is a physician-scientist whose work is focused on the pathogenesis of Crohn's disease. She completed her MD and PhD degrees as well as her internal medicine residency and gastroenterology fellowship at the University of Washington (UW). After fellowship, she obtained an NIH K award to start her own lab and joined the faculty there, where she was for the past 8 years. Mary's early career was very productive. She successfully applied for two R01 grants and built a lab team at UW consisting of three graduate students, three postdoctoral fellows, a research assistant, and a lab manager. Her group has published several high-impact papers in the past few years, including a major breakthrough in *Cell*, which brought Mary national recognition as a leader in the Crohn's disease field. Mary recently accepted a position as the director of the Division of Gastroenterology in the Department of Medicine at the University of California, San Francisco (UCSF). She was invited to interview for the position last year after her promotion to associate professor at UW. She had been intrigued by the idea of becoming a division director, but her prospects at UW were limited. The GI division director there was a recent recruit and widely viewed as doing an excellent job. The UCSF GI division director position was a terrific opportunity for her, as that division is considered one of the best in the country. They have an excellent group in inflammatory bowel disease with whom Mary knew she would love to work. Before interviewing, Mary discussed the possibility of moving with her husband Tim, a patent attorney with Microsoft. Tim was equally enthusiastic about the potential move, and they considered together how the decision would affect their 12- and 14-year-old daughters. They suspected that the girls probably wouldn't be thrilled, but they also knew that they wouldn't have a problem integrating quickly into a new school and social environment. Mary's interview visit went very well. Soon after her return home, she received a formal job offer from UCSF. The offer included a substantial raise (almost double her salary), a $2.5 million startup package for her lab and an additional $5 million to recruit new faculty members to the division over the next 5 years. After a second visit to UCSF, this time with Tim and the girls, Mary accepted the offer. She made the official move to UCSF with her family last month, after the girls finished their school year. Mary thought that her lab team would be equally excited about the move. Her lab at UCSF is in a new building with a great view of the city, and there are plenty of resources to quickly move projects forward. However, her two senior students at UW were 6–12 months away from graduating and had no interest in moving. Her new graduate student was frustrated about the current direction of his project and decided to switch labs and start over. Of her three postdocs, one had been with her for 4 years and was ready to start looking for an independent position. Two others had just started in the lab and strongly considered looking for other positions in Seattle. Ultimately, only her lab manager and one of her new postdocs were willing to move to UCSF. Mary is currently in the process of reestablishing her lab team. Just last week, Mary learned that the interim division director at UCSF, a superb clinical gastroenterologist, is leaving soon to take a leadership position at a competing hospital across town. He is loved in the division and may take a large group of the GI nursing staff with him. To complicate matters, Mary received a report today of the third incident of a serious error during endoscopy by a senior fellow in the GI division. The recommendation is that the fellow be dismissed, but the final decision will be hers to make. After dinner, Mary recounts these events to Tim while the girls are helping clear the dishes. He opens his laptop to schedule his testing date for the California bar exam and tries to listen patiently. Alice, their older daughter, says as she walks past, ‘Why did we have to move, anyway? You're making me start high school in a new place where I don't have *any* friends. I wish we could just go home’. Mary then hears the familiar ping of an incoming email on her phone and glances briefly to find a note from her department chair requesting a proposal for a new pancreas/small intestine/liver transplant program to be instituted at UCSF. He is meeting with a benefactor over dinner tomorrow who is willing to give millions of dollars to the effort, and he needs a well-organized plan to make a good pitch for the program. She sighs and adds one more thing to her to-do list for the evening. Discussion questions:

1. Should Mary have taken on this new leadership role?
2. How should she recruit new lab members at UCSF?
3. What should Mary's expectations be for her first year in the new position?
4. Given so many demands on her time, what should be her top priorities?
5. Who are her mentors in her new role as division director?
